# Effects of *Ligilactobacillus agilis* W70 and dietary protein levels on feed efficiency, nitrogen metabolism, and rumen microbiota of lactating dairy cows

**DOI:** 10.1186/s42523-026-00548-7

**Published:** 2026-03-24

**Authors:** Xiaowei Duan, Lu Ma, Calum Bridson, Zhijie Luo, Stafford Vigors, Dengpan Bu

**Affiliations:** 1 State Key Laboratory of Animal Nutrition and Feeding, Institute of Animal Science, Chinese Academy of Agricultural Sciences, Beijing, 100193 China; 2 School of Agriculture and Food Science, University College Dublin, Dublin, D04V1W8 Ireland; 3 National Center of Technology Innovation for Dairy, Inner Mongolia, China; 4 China-Ireland Dairy Sustainable Development Center, Beijing, 100193 China

**Keywords:** Probiotic supplementation, Nitrogen utilization, Microbial community, Milk production, Sustainable agriculture

## Abstract

**Background:**

Efficient nitrogen utilization in dairy cows is essential for maximizing lactation performance while minimizing nitrogen losses. This experiment was conducted to evaluate the effects of the additive W70 and varying dietary protein levels on lactation performance, rumen fermentation, blood parameters, and nitrogen metabolism in lactating dairy cows.

**Method:**

16 Holstein cows were utilised in a replicated 4 × 4 Latin square with 2 levels of crude protein (CP) (16 vs. 17%) and 2 levels of additive W70 (0 vs. 5 × 10^10^ cfu/g, 20 g/d). The cows were randomly assigned to 4 treatment groups: 16% CP no additive (16%C), 16% CP with additive W70 (16%A), 17% CP no additive (17%C), 17% CP with additive W70 (17%A).

**Results:**

An interaction was observed between CP and additive W70. Specifically, additive W70 decreased dry matter intake (DMI) (*P* < 0.01), nitrogen (N) intake (*P* < 0.01), retained N content (*P* < 0.05), productive N content (*P* < 0.05), retained N proportion (*P* < 0.05), productive N proportion (*P* < 0.10), increased milk yield/DMI (*P* < 0.01) and nitrogen utilization efficiency (NUE) (*P* < 0.10) in the 16% CP diet, whereas no differences were observed in the 17% CP diet. The additive W70 increased fat corrected milk (FCM)/DMI (*P* < 0.10) and energy corrected milk (ECM)/DMI (*P* < 0.05), and reduced ruminal ammonia nitrogen (NH_3_-N) (*P* < 0.01), with no interaction with dietary CP level. The relative abundance of the genera *Sphingomonas*,* Solibacillus*,* Desemzia*,* Peribacillus*,* Succinivibrionaceae UGG-001*,* Prevotella*, and *Succiniclasticum* were higher in the rumen following the addition of additive W70 in both the 16% and 17% CP diets. Milk yield (*P* < 0.01), milk urea nitrogen (MUN) (*P* < 0.01), milk protein yield (*P* < 0.01), FCM yield (*P* < 0.05), ECM yield (*P* < 0.01), FCM/DMI (*P* < 0.10), and ECM/DMI (*P* < 0.05) were all increased in response to the 17% CP diet, whereas DMI and the content of milk fat, milk protein, and milk lactose were unaffected. The 17% CP diet increased the acetate to propionate ratio (*P* < 0.10) and NH_3_-N (*P* < 0.10), while reducing isovalerate (*P* < 0.10) and valerate (*P* < 0.10). The 17% CP diet increased blood urea nitrogen (BUN) (*P* < 0.01) and glucose (GLU) (*P* < 0.05). Cows fed a 17% CP diet had a higher milk N content (*P* < 0.05), fecal N content (*P* < 0.01), N loss content (*P* < 0.01), fecal N proportion (*P* < 0.01), urine N proportion (*P* < 0.10), and N loss proportion (*P* < 0.10) than those receiving 16% CP. The relative abundance of the genera *Kandleria*,* Ligilactobacillus*, and *Escherichia-Shigella* were lower in the 17% CP diet than in the 16% CP diet despite whether additive W70 was added or not.

**Conclusion:**

Supplementation with additive W70 improved feed conversion and NUE in a 16% CP diet, while the additive W70 modulated the rumen microbiota and reduced ruminal NH_3_-N. The 17% CP diet enhanced milk yield but increased N losses and altered rumen microbial composition.

**Supplementary Information:**

The online version contains supplementary material available at 10.1186/s42523-026-00548-7.

## Introduction

Nitrogen metabolism in lactating dairy cows is a crucial determinant of both milk production and the subsequent nutrient excretion, however, NUE in ruminants is on average only around 25% [1, 2]. Inefficient nitrogen utilization can lead to excessive N excretion in the form of urea [3]. Enhancing NUE in dairy cows not only improves milk yield and composition but also reduces the environmental impact of dairy farming [4].

Direct-fed microbials (DFM) have been widely studied for their beneficial effects on gut health and nutrient absorption in various animal species [5]. In ruminants, DFM can modulate rumen fermentation patterns, improve feed efficiency, and enhance nutrient utilization [6]. Lactic acid bacteria (LAB) are known to enhance rumen fermentation and nutrient utilization by producing metabolites such as volatile fatty acids (VFAs) [7], which serve as additional energy sources for the host animal, thereby supporting productivity and health [8]. Previous studies have reported that certain ruminal ammonia-utilizing microorganisms activate the ammonia utilization pathway, participate in the ruminal ammonia assimilation process [9], and promote the synthesis of amino acids (AA) and microbial protein (MCP) [10], thereby enhancing NUE [11]. Additive W70 is a lactic acid producing bacteria and a probiotic with the capacity to enhance ammonia assimilation in the rumen [12]. In addition, dietary protein levels can also influence nitrogen metabolism in dairy cows [13]. High-protein diets provide more nitrogen for production, but can lead to increased nitrogen excretion if not efficiently utilized [14]. Conversely, low-protein diets can limit microbial protein synthesis due to insufficient ammonia availability [15]. Previous studies reported that balancing dietary protein levels with DFM supplementation can optimize nitrogen utilization [16]. However, there is a research gap in verifying nitrogen utilization efficiency by using strains with dual functions of ammonia utilization and lactate production in different protein diets.

Previous studies have demonstrated that DFM can improve NUE in dairy cows, although most of this research has primarily focused on *Saccharomyces cerevisiae* [17]. Additive W70, an indigenous strain isolated from the rumen [18], has the capability to assimilate ammonia and enhance rumen fermentation [12]. This study aims to evaluate the effects of additive W70 on lactation performance, rumen fermentation, blood indicators, nitrogen metabolism, and rumen microbiota in lactating dairy cows fed diets with 16% or 17% CP. We hypothesize that additive W70 supplementation will enhance lactation performance and NUE in both 16% CP and 17% CP diets by improving ruminal fermentation, enhancing microbial protein synthesis, and regulating rumen microbiota. The findings from this research could lead to more effective dietary strategies for dairy cows, combining additive W70 and optimized protein levels to achieve both productive and environmental benefits.

## Results

### The effect of dietary protein and additive W70 on lactation performance

There was a protein × additive interaction effect for DMI (*P* < 0.01) and Milk yield/DMI (*P* < 0.01) of lactating dairy cows (Table 1) where DMI was lower with the additive than without it in the 16% CP diet, while no difference was identified in cows fed the 17% CP diet (*P* < 0.01). In contrast, Milk yield/DMI was higher with the additive than without it in the 16% CP diet, while no difference was found in cows fed the 17% CP diet (*P* < 0.01).

**Table 1:** Dry matter intake and lactation performance of lactating dairy cows fed different dietary protein level (16%/17%) or diets supplemented with additive W70 (C/A)

Item	Treatment^1^				SEM	*P* Value^2^		
	16%C	16%A	17%C	17%A		Protein	Additive	*P*×A
DMI^3^ (kg/d)	22.20	20.31	23.45	23.12	0.43	**< 0.01**	**< 0.01**	**< 0.01**
Production (kg/d)								
Milk yield	28.90	28.80	29.69	30.12	0.87	**< 0.01**	0.60	0.26
Fat	1.40	1.31	1.38	1.44	0.07	0.31	0.69	0.16
Protein	1.12	1.10	1.15	1.17	0.07	**< 0.01**	0.86	0.13
Lactose	1.67	1.65	1.69	1.70	0.11	0.10	0.95	0.20
3.5% FCM^3^	35.28	33.71	36.58	36.31	1.27	**0.02**	0.28	0.35
ECM^3^	35.72	34.35	36.89	36.93	1.15	**0.01**	0.36	0.25
Composition (%)								
Fat	4.77	4.44	4.69	4.67	0.19	0.60	0.22	0.29
Protein	3.84	3.80	3.87	3.87	0.05	0.19	0.65	0.48
Lactose	4.35	4.32	4.32	4.31	0.05	0.32	0.38	0.38
Total Solids	15.52	15.09	15.57	15.43	0.30	0.27	0.12	0.37
Somatic cell count (10^4^ cell/mL)	23.75	30.21	22.34	22.27	5.90	0.37	0.54	0.42
MUN^3^ (mg/dL)	15.09	15.61	16.56	16.63	0.59	**0.01**	0.54	0.63
Efficiency								
Milk yield/DMI	1.29	1.43	1.26	1.31	0.02	**< 0.01**	**< 0.01**	**< 0.01**
FCM/DMI	1.66	1.74	1.62	1.66	0.04	**0.08**	**0.08**	0.37
ECM/DMI	1.86	1.96	1.83	1.87	0.04	**0.04**	**0.02**	0.25

^1^16%C = 16% CP no additive, 16%A = 16% CP with additive W70 (5 × 10^10^ cfu/g, 20 g/d), 17%C = 17% CP no additive, 17%A = 17% CP with additive W70 (5 × 10^10^ cfu/g, 20 g/d)

^2^Protein = main effect of dietary protein level; Additive = main effect of additive W70; P × A = interaction of dietary protein level and additive W70

^3^DMI = dry matter intake, 3.5% FCM (fat corrected milk) (kg/d) = 0.4324 × milk (kg/d) + 16.216 × fat (kg/d), ECM (energy corrected milk) (kg/d) = 0.327 × milk (kg/d) + 12.95 × fat (kg/d) + 7.65 × protein (kg/d), MUN = milk urea nitrogen

The addition of additive W70 improved ECM/DMI (*P* < 0.10) and tended to improve FCM/DMI (*P* < 0.10). The 17% CP diet increased milk yield (*P* < 0.01), milk protein yield (*P* < 0.01), 3.5% FCM yield (*P* < 0.05), ECM yield (*P* < 0.05), MUN (*P* < 0.05), FCM/DMI (*P* < 0.10), and ECM/DMI (*P* < 0.05).

### The effect of dietary protein and additive W70 on rumen fermentation

There was a protein × additive interaction effect for rumen pH (*P* < 0.01) of lactating dairy cows (Table 2). The pH was higher in cows receiving the additive on the 16% CP diet when compared with cows not receiving the additive, while pH was lower in the cows receiving the additive on the 17% CP diet when compared with cows receiving no additive (*P* < 0.01). The addition of additive W70 reduced ruminal NH_3_-N (*P* < 0.01). The 17% CP diet showed a tendency to reduce isovalerate (*P* < 0.10) and valerate (*P* < 0.10), increase the acetate-propionate ratio (*P* < 0.10), and NH_3_-N (*P* < 0.10).

**Table 2:** Rumen fermentation of lactating dairy cows fed different dietary protein level (16%/17%) or diets supplemented with additive W70 (C/A)

Item	Treatment^1^				SEM	*P* Value^2^		
	16%C	16%A	17%C	17%A		Protein	Additive	*P*×A
pH	6.74	6.88	6.97	6.83	0.06	**0.05**	0.97	**< 0.01**
Total VFAs^3^ (mmol/L)	78.71	82.26	77.90	75.20	4.59	0.31	0.91	0.42
Acetate (mmol/L)	48.68	50.78	49.38	47.97	2.41	0.61	0.87	0.40
Propionate (mmol/L)	17.87	18.70	16.89	16.27	1.55	0.17	0.93	0.56
Iso-butyrate (mmol/L)	0.71	0.75	0.74	0.70	0.05	0.81	1.00	0.41
Butyrate (mmol/L)	9.11	9.47	8.67	8.21	0.73	0.20	0.94	0.54
Isovalerate (mmol/L)	1.22	1.35	1.19	1.07	0.09	**0.07**	0.99	0.13
Valerate (mmol/L)	1.12	1.22	1.03	0.98	0.10	**0.07**	0.77	0.40
A: P^3^	2.81	2.98	3.13	3.19	0.17	**0.07**	0.41	0.69
Methane^4^	20.61	21.50	21.04	20.40	1.02	0.70	0.89	0.37
NH_3_-N (mg/dL)	12.64	9.50	13.23	11.29	0.98	**0.09**	**< 0.01**	0.39
MCP^3^ (g/L)	0.67	0.63	0.70	0.63	0.04	0.58	0.13	0.67

^1^16%C = 16% CP no additive, 16%A = 16% CP with additive W70 (5 × 10^10^ cfu/g, 20 g/d), 17%C = 17% CP no additive, 17%A = 17% CP with additive W70 (5 × 10^10^ cfu/g, 20 g/d)

^2^Protein = main effect of dietary protein level; Additive = main effect of additive W70; P × A = interaction of dietary protein level and additive W70

^3^VFAs = Volatile Fatty Acids, A:P = the ratio of acetate and propionate, MCP = microbial protein

^4^Estimated from stoichiometric calculation using the following equation: Methane = 0.45 × acetate (mol/100 mol) − 0.275 × propionate (mol/100 mol) + 0.40 × butyrate (mol/100 mol) (Moss et al., 2000)

### The effect of dietary protein and additive W70 on blood indicators

There was no protein × additive interaction effect for blood chemistry indexes of lactating dairy cows (Table 3). The addition of W70 had no effect on blood indicators. The 17% CP diet increased the levels of BUN (*P* < 0.01) and GLU (*P* < 0.05) when compared to cows fed the 16% CP diet.

**Table 3:** Blood chemistry parameters of lactating dairy cows fed different dietary protein level (16%/17%) or diets supplemented with additive W70 (C/A)

Item	Treatment^1^				SEM	*P* Value^2^		
	16%C	16%A	17%C	17%A		Protein	Additive	*P*×A
TP^3^ (g/L)	80.49	78.37	80.16	81.34	2.44	0.59	0.85	0.50
ALB^3^ (g/L)	38.09	38.07	37.44	38.08	0.54	0.42	0.43	0.40
GLB^3^ (g/L)	42.40	40.31	42.73	43.26	2.26	0.47	0.73	0.56
ALB/GLB	0.96	1.02	0.91	0.90	0.05	0.10	0.56	0.47
BUN^3^ (mmol/L)	4.04	4.24	4.67	4.98	0.23	**< 0.01**	0.13	0.75
GLU^3^ (mmol/L)	1.35	1.47	1.72	1.70	0.13	**0.02**	0.65	0.56
T-CHO^3^ (mmol/L)	6.28	6.21	6.34	6.34	0.32	0.48	0.79	0.78
TG^3^ (mmol/L)	0.15	0.15	0.15	0.15	0.01	0.99	0.85	0.61
ALT^3^ (U/mL)	2.96	2.93	2.97	2.94	0.09	0.89	0.65	0.90
AST^3^ (U/mL)	1.28	1.16	1.08	1.11	0.09	0.12	0.57	0.33

^1^16%C = 16% CP no additive, 16%A = 16% CP with additive W70 (5 × 10^10^ cfu/g, 20 g/d), 17%C = 17% CP no additive, 17%A = 17% CP with additive W70 (5 × 10^10^ cfu/g, 20 g/d)

^2^Protein = main effect of dietary protein level; Additive = main effect of additive *L. agilis* W70; P × A = interaction of dietary protein level and additive W70

^3^TP = total protein, ALB = albumin, GLB = globulins, BUN = blood urea nitrogen, GLU = glucose, T-CHO = total cholesterol, TG = triglycerides, ALT = alanine aminotransferase, AST = aspartate aminotransferase

### The effect of dietary protein and additive W70 on nitrogen metabolism

There was a protein × additive interaction effect for N intake (*P* < 0.01), retained N content (*P* < 0.05), productive N content (*P* < 0.05), retained N proportion (*P* < 0.05), productive N proportion (*P* < 0.10), and NUE (*P* < 0.10) of lactating dairy cows (Table 4). N intake was lower in cows fed the additive when compared to cows receiving no additive on the 16% CP diet, while no difference was identified in cows fed the 17% CP diet (*P* < 0.01). In contrast, retained N content (*P* < 0.05), productive N content (*P* < 0.05), retained N proportion (*P* < 0.05), productive N proportion (*P* < 0.10), and NUE (*P* < 0.10) was higher in cows fed the additive when compared to cows receiving no additive on the 16% CP diet, while no difference was identified in cows fed the 17% CP diet. Feeding the 17% CP diet increased milk N content (*P* < 0.05), fecal N content (*P* < 0.01), N loss content (*P* < 0.01), fecal N proportion (*P* < 0.01), urine N proportion (*P* < 0.10), N loss proportion (*P* < 0.10). The predicted supply of metabolizable protein (MP) and AA from the dietary treatments is shown in Table 5. Milk protein synthesis was enhanced by the 17% CP diet due to increased AA availability as shown in Table 5.

**Table 4:** Nitrogen metabolism of lactating dairy cows fed different dietary protein level (16%/17%) or diets supplemented with additive W70 (C/A)

Item	Treatment^1^				SEM	*P* Value^2^		
	16%C	16%A	17%C	17%A		Protein	Additive	*P*×A
N Intake (g/d)	581.04	535.63	641.71	637.91	11.91	**< 0.01**	**< 0.01**	**< 0.01**
Milk N (g/d)	178.36	178.46	185.97	188.48	6.93	**0.01**	0.68	0.70
Fecal N (g/d)	89.30	85.46	139.85	140.48	7.19	**< 0.01**	0.81	0.73
Urine N (g/d)	162.92	160.99	180.45	156.75	10.40	0.51	0.21	0.29
Loss N^3^ (g/d)	252.22	246.46	320.30	297.23	11.43	**< 0.01**	0.21	0.45
Retained N^3^ (g/d)	150.46	110.72	135.43	152.20	11.85	0.27	0.33	**0.02**
Productive N^3^ (g/d)	328.81	289.18	321.41	340.68	13.40	**0.08**	0.42	**0.02**
Fecal N (%)	15.37	15.95	21.96	21.92	1.22	**< 0.01**	0.81	0.79
Urine N (%)	28.39	30.09	27.89	24.52	1.66	**0.07**	0.61	0.13
Loss N (%)	43.76	46.04	49.85	46.44	1.71	**0.06**	0.74	0.10
Retained N (%)	25.51	20.21	21.26	23.54	1.80	0.80	0.41	**0.04**
Productive N (%)	56.24	53.96	50.15	53.56	1.71	**0.06**	0.74	**0.08**
NUE^3^	30.73	33.75	28.89	30.02	0.75	**< 0.01**	**< 0.01**	**0.06**

^1^16%C = 16% CP no additive, 16%A = 16% CP with additive W70 (5 × 10^10^ cfu/g, 20 g/d), 17%C = 17% CP no additive, 17%A = 17% CP with additive W70 (5 × 10^10^ cfu/g, 20 g/d)

^2^Protein = main effect of dietary protein level; Additive = main effect of additive *L. agilis* W70; P × A = interaction of dietary protein level and additive W70

^3^Loss N = Fecal N + Urine N, Retained N = N Intake - Loss N - Milk N, Productive N = Milk N + Retained N, NUE = nitrogen utilization efficiency

**Table 5:** Predicted supply of metabolizable protein (MP) and amino acids (AA) from dietary treatments by NASEM, 2021

Item	Predicted Supply g/d				Target Metab AA Efficiency		Predicted Metab AA Efficiency		Predicted Milk Protein g/d	
	16%^1^	MP,%	17%^1^	MP,%	16%^1^	17%^1^	16%^1^	17%^1^	16%^1^	17%^1^
Arg	135	5.65	143	5.71	-	-	0.48	0.45	0	0
His	57	2.38	60	2.4	0.75	0.75	0.82	0.79	94	99
Ile	137	5.73	144	5.75	0.71	0.71	0.66	0.63	120	126
Leu	214	8.95	222	8.87	0.73	0.73	0.73	0.71	98	102
Lys	175	7.32	185	7.39	0.72	0.72	0.75	0.71	198	209
Met	53	2.22	55	2.2	0.73	0.73	0.79	0.77	96	0
Phe	134	5.6	140	5.59	0.6	0.6	0.59	0.57	0	0
Thr	125	5.23	131	5.23	0.64	0.64	0.63	0.6	0	0
Trp	31	1.3	33	1.32	0.86	0.86	0.82	0.78	0	0
Val	146	6.11	153	6.11	0.74	0.74	0.72	0.69	0	0
EAA^2^	1208	50.52	1267	50.62	-	-	0.68	0.65	-149	-163
Other AA^3^	2114	88.41	2213	88.41	-	-	-	-	163	171

^1^16% = 16% CP diet, 17%= 17% CP diet

^2^EAA = essential amino acids, included Arg, His, Ile, Leu, Lys, Met, Phe, Thr, Trp, and Val [104]

^3^Other AA = non-essential amino acids (NEAAs), included conditional essential amino acids

### The effect of dietary protein and additive W70 on rumen microbiota

**Fig. 1:**
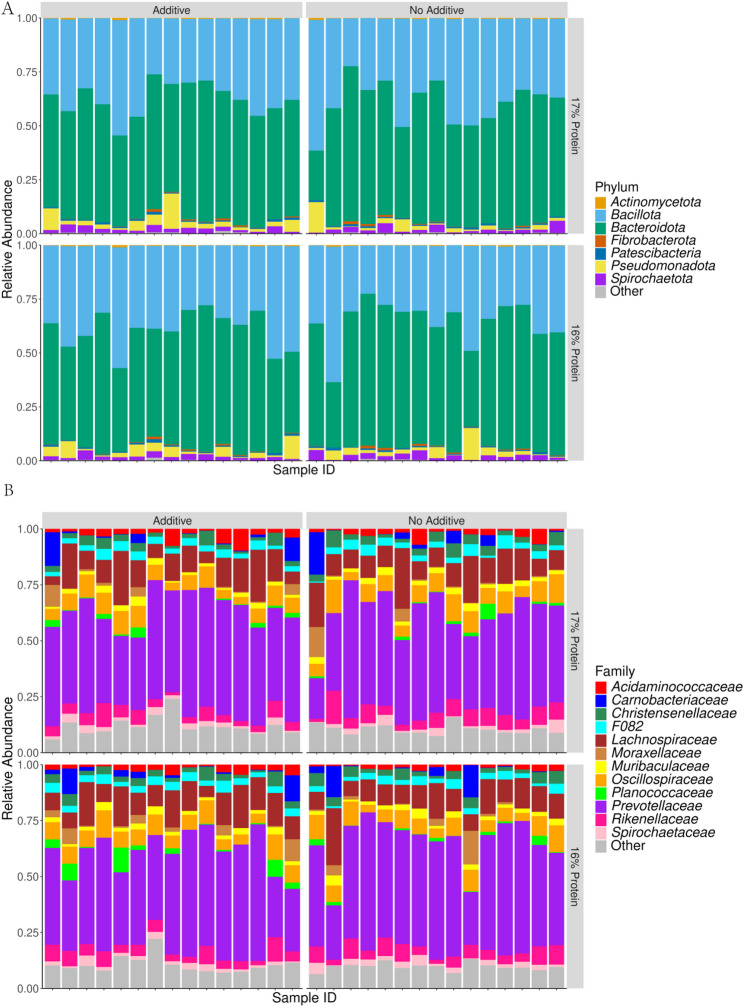
Histogram of clustering of phylum **(A)**, and genera **(B)** abundance in main effects Additive **(A-C)** and Protein (16%-17%). (A = additive W70, C = no additive, 16% = 16% CP diet, 17% = 17% CP diet)

**Fig. 2:**
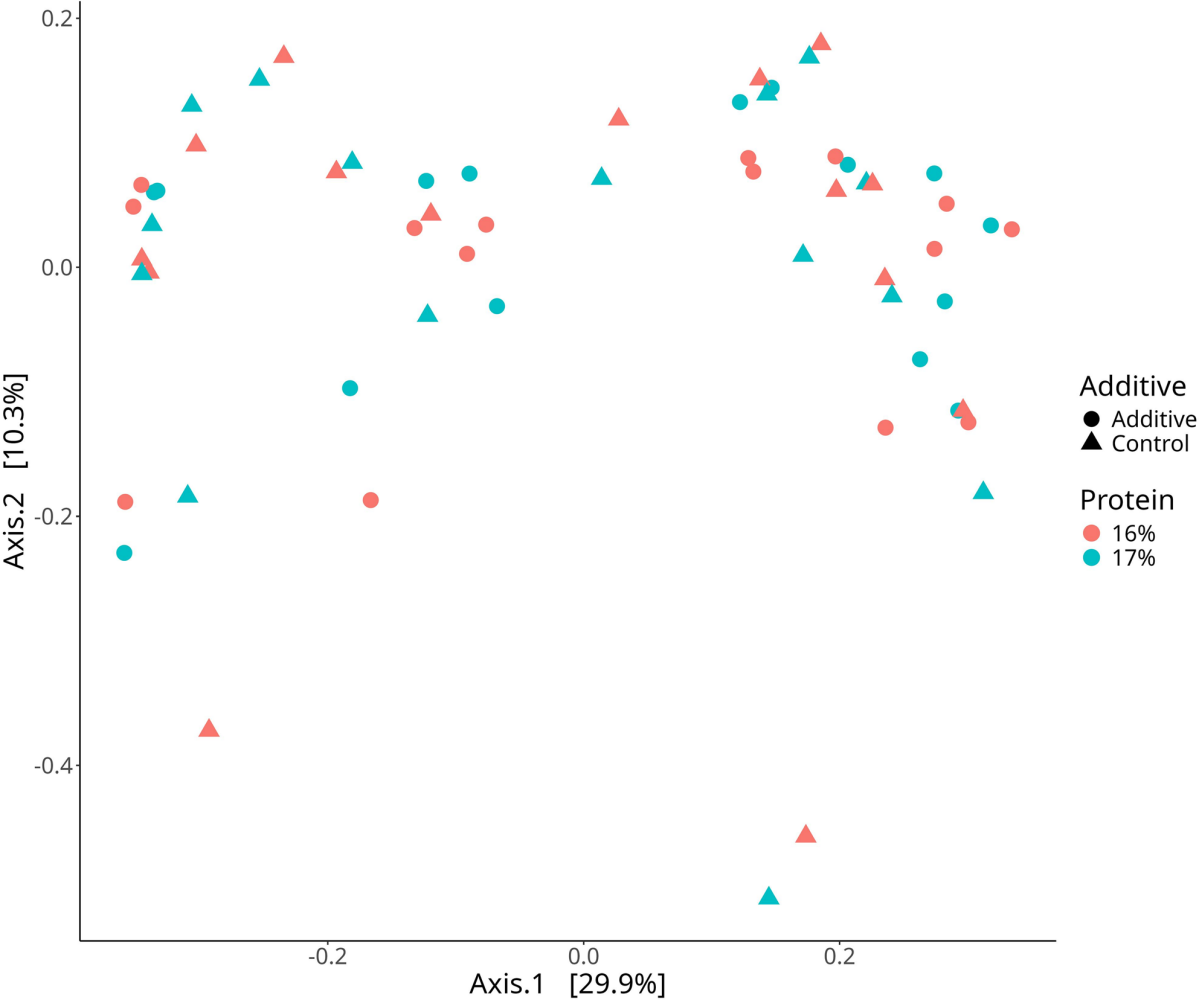
PCoA community composition in main effects Additive (**A**-**C**) and Protein (16%-17%). (A = additive W70, C = no additive, 16% = 16% CP diet, 17% = 17% CP diet)

**Fig. 3:**
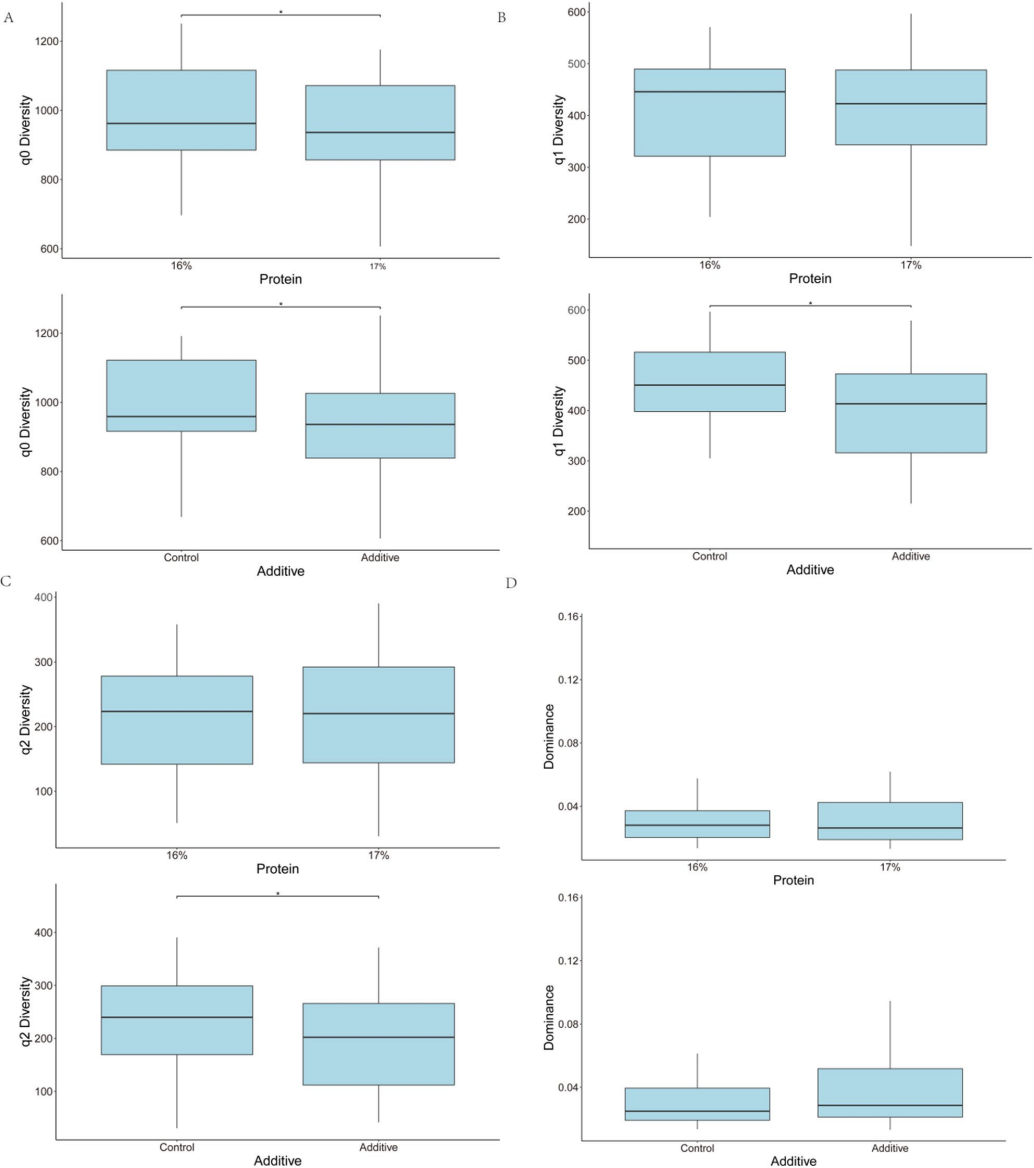
Alpha diversity differences of q0 **(A)**, q1 **(B)**, q2 **(C)**, and dominance **(D)** in main effects Additive (A-C) and Protein (16%-17%). (q0 = species richness, q1 = Shannon’s index, q2 = inverse Simpson’s index, A = additive W70, C = no additive, 16% = 16% CP diet, 17% = 17% CP diet)

**Fig. 4:**
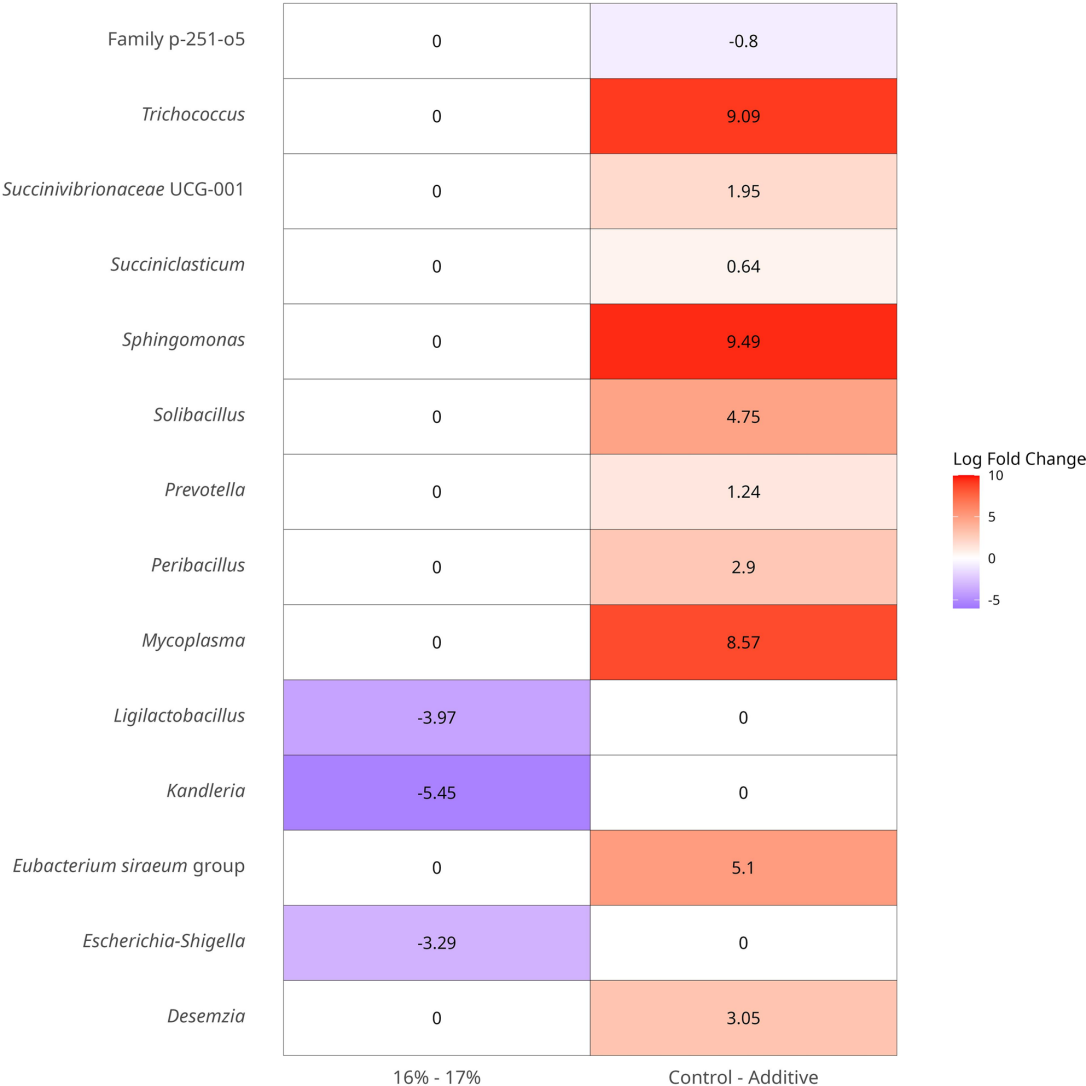
DESeq2 analysis of relative abundance differences (adjusted *P*-value < 0.05) at the genera level of the main effects of Additive (**A**-**C**) and Protein (16%-17%). (Negative log fold change estimates correspond to reduced abundance of each genera in the 16% (left plots) and Control (right plots)) (A = additive W70, C = no additive, 16% = 16% CP diet, 17% = 17% CP diet)

**Fig. 5:**
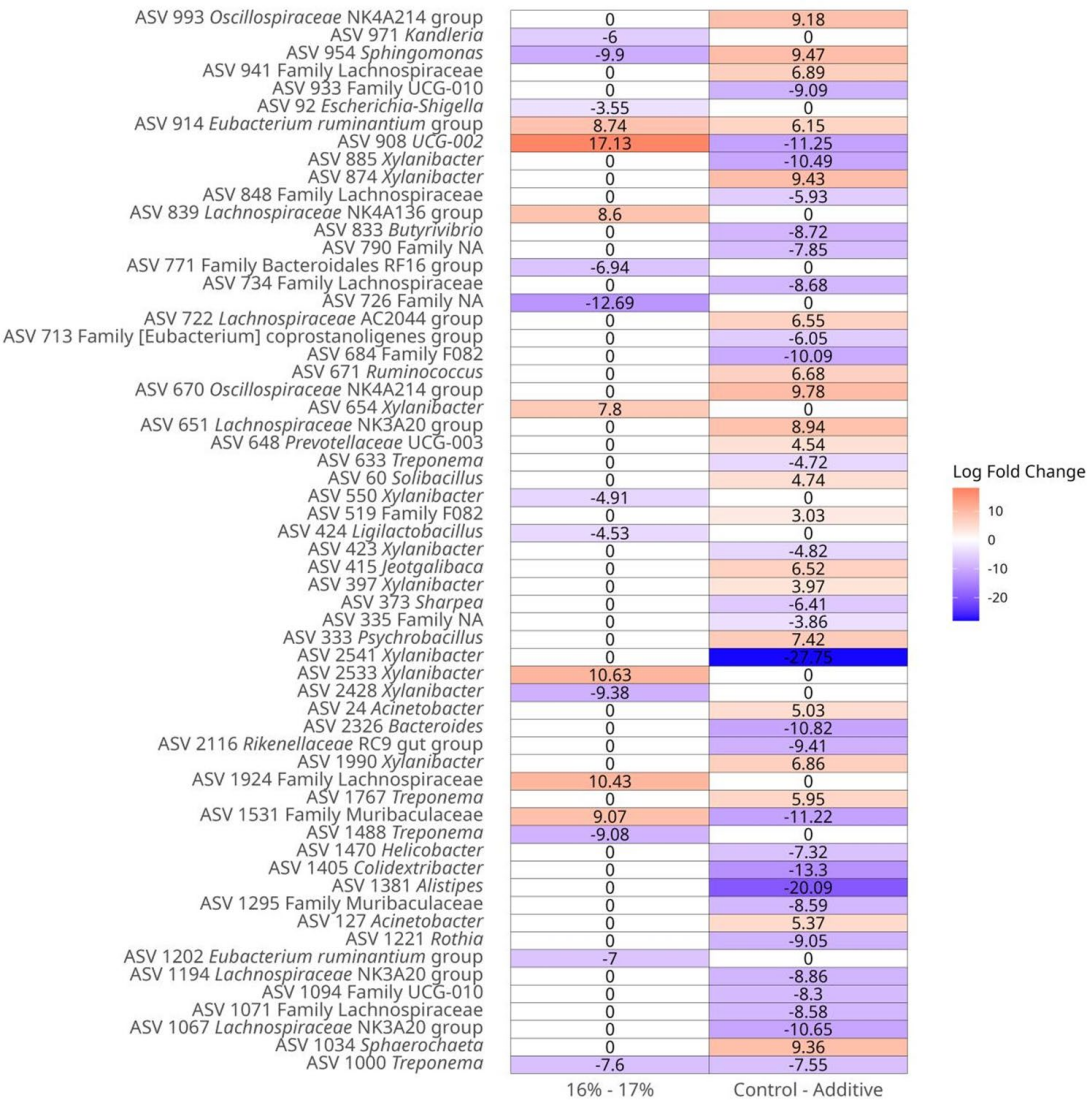
DESeq2 analysis of relative abundance differences (adjusted *P*-value < 0.05) at the ASV level in main effects Additive **(A-C)** and Protein (16%-17%). (Negative log fold change estimates correspond to reduced abundance of each ASV in the 16% (left plots) and Control (right plots)) (A = additive W70, C = no additive, 16% = 16% CP diet, 17% = 17% CP diet)

The rarefaction curves of all 60 Samples are presented in Fig. S1. The histogram of clustering of phylum, family, and genera relative abundance for the main effects of Additive (A-C) and Protein (16%-17%) are shown in Fig. 1A, Fig. S2, and Fig. 1B respectively. Across all samples irrespective of treatment, the composition at phylum level was 55.00% Bacteroidota, 38.20% Bacillota, 3.19% Pseudomonadota, 1.97% Spirochaetota, 0.71% Patescibacteria, and 0.33% Fibrobacterota. Across all samples irrespective of treatment, the composition at family level was 42.50% *Prevotellaceae*, 11.90% *Lachnospiraceae*, 7.32% *Oscillospiraceae*, 5.28% *Rikenellaceae*, 3.24% *Christensenellaceae*, 2.94% F082, 2.38% *Acidaminococcaceae*, 2.32% *Carnobacteriaceae*, 2.09% *Muribaculaceae*, 1.97% *Spirochaetaceae*, 1.76% *Moraxellaceae*. Across all samples irrespective of treatment, the composition genera level was 32.90% *Xylanibacter*, 5.08% *Rikenellaceae RC9* gut group, 4.07% *NK4A214* group, 3.27% *Prevotella*, 2.92% *Christensenellaceae* R-7 group, 2.36% *Succiniclasticum*, 2.11% *Carnobacterium*, 2.01% *Shuttleworthia*, 1.94% *Lachnospiraceae NK3A20* group, 1.93% *Treponema*, 1.53% *Prevotellaceae* UGG-001. The PCoA Community Composition identified no separation based on the main effects of A-C and 16%-17% (Fig. 2). The alpha diversity metrics (species richness, Shannon’s index, inverse Simpson’s index, and dominance) for the comparison of main effects A-C and 16%-17% are presented in Fig. 3. For the comparison of A-C (Fig. 3A, B and C), the C fed cows had higher species richness (*P* < 0.05), Shannon’s index (*P* < 0.05), and inverse Simpson’s index (*P* < 0.05) compared to the A group. The relative abundance differences at the family level for the main effects of A-C and 16%-17% evaluated using DESeq2 are presented in Fig. S3. Negative log fold change estimates correspond to reduced abundance of each amplicon sequence variant (ASV) in the 16% and Control in the left and right plots, respectively. In contrast, positive log fold change estimates correspond to increased abundance of each ASV in in the 17% and Additive in the left and right plots, respectively. The relative abundance of the families *Sphingomonadaceae* (0.028% vs. 0.007%), *Mycoplasmataceae* (0.014% vs. 0.007%), *Bacillaceae* (0.148% vs. 0.022%), *Succinivibrionaceae* (1.067% vs. 0.24%), *Acidaminococcaceae* (2.835% vs. 1.984), and *Selenomonadaceae* (1.330% vs. 0.876%) were higher in A than C, and the relative abundance of the family p-251-o5 (0.211% vs. 0.306%) was lower in A than C, according to DESeq2 (Fig. S3). The relative abundance of the family *Enterobacteriaceae* was lower in the 17% group than the 16%. The relative abundance differences at the genera level of effects A-C and 16%-17% are shown in Fig. 4. The relative abundance of the genera *Sphingomonas* (0.028% vs. 0.007%), *Solibacillus* (0.65% vs. 0.095%), *Desemzia* (0.104% vs. 0.100%), *Peribacillus* (0.147% vs. 0.020%), *Succinivibrionaceae UGG-001* (0.974% vs. 0.127%), *Prevotella* (3.993% vs. 2.584%), and *Succiniclasticum* (2.811% vs. 1.976%) were higher in A than C, and the relative abundance of the Family p-251-o5 (0.211% vs. 0.306) was lower in A than C (Fig. 4). The relative abundance of the genus *Kandleria* (0.014% vs. 0.017%), *Ligilactobacillus* (0.052% vs. 0.056%), and *Escherichia-Shigella* were lower in the 17% group than 16%. The relative abundance differences at the ASV level of the main effects of A-C and 16%-17% are presented in Fig. 5.

Correlation analysis between rumen fluid microbiota and rumen fermentation parameters in main effects additive (A-C) are presented in Fig. S4. In A group, NH_3_-N was negatively associated with *Acinetobacter* (*P* < 0.05), while isobutyrate and total VFAs were positively associated with F082 (*P* < 0.05). MCP levels were negatively associated with *Acinetobacter* (*P* < 0.05) and *Cornobacterium* (*P* < 0.01) in A group. In C group, pH was positively associated with *Acinetobacter* (*P* < 0.05), *Carnobacterium* (*P* < 0.05), and *Colidextribacter* (*P* < 0.05), but negatively associated with the *Bacteroidales* RF16 group (*P* < 0.05), F082 (*P* < 0.05), *Prevotellaceae* Ga6A1 group (*P* < 0.05), *Prevotellaceae* UCG-004 (*P* < 0.001), and *Treponema* (*P* < 0.05). Additionally, there was a negative association between total VFAs (*P* < 0.05) and *Selenomonadaceae* (*P* < 0.05) in A group. Correlation analysis between rumen fluid microbiota and rumen fermentation parameters in the comparison of protein (16%-17%) are presented in Fig. S5. The 17% group showed negative associations of NH_3_-N with *Acinetobacter* (*P* < 0.05), of the AP ratio with NK4A214_group (*P* < 0.05), and of isobutyrate with *Burkholderia Caballeroinia Paraburkholderia* (*P* < 0.05). The 16% group showed that pH was positively associated with *Colidextribacter* (*P* < 0.01) and negatively associated with *Prevotellaceae* UCG-004 (*P* < 0.05) and *Selenomonadaceae* (*P* < 0.05), while Total VFAs were positively associated with F082 (*P* < 0.05).

## Discussion

### The effect of additive W70 and dietary protein on lactation performance

This experiment was conducted to evaluate the effects of additive W70 and varying dietary protein levels on lactation performance, rumen fermentation, blood parameters, and nitrogen metabolism in lactating dairy cows.

An interaction between dietary protein level and additive was observed for DMI and milk yield/DMI, indicating that the additive’s effect varied based on the level of dietary protein, suggesting that the additive decreased the DMI and more effectively improved feed efficiency under low-protein conditions in dairy cows. The effects of probiotics on DMI vary across studies, with some reporting increases due to improved rumen fermentation [19], while others found no changes with probiotic supplementation [20]. In this study, the reduction in DMI with the additive W70 [12] might be attributed to LAB metabolites (e.g., lactate, acetate, formate, propionate), which can influence satiety [21] and metabolic signals such as propionate-induced hypophagia [22]. The additive W70 had no effect on milk yield or the concentrations of milk fat, protein, lactose, or total solids. This is consistent with the observed unchanged levels of acetate and propionate in the rumen, key drivers of milk fat, lactose, and protein synthesis [23–25]. Probiotic supplementation has been associated with increases in ECM and FCM [26, 27], but these effects are often dose-dependent. For example, microbial feed supplements improved ECM in cows with lower baseline ECM but had no effect or negative impacts at higher ECM thresholds [28]. In this study, ECM and FCM remained unchanged by additive W70, likely due to unchanged milk yield and composition. While Schofield et al. [29] demonstrated that probiotics improve feed efficiency by enhancing nutrient absorption and rumen fermentation, the improvement in feed efficiency (Milk yield/DMI, FCM/DMI, and ECM/DMI) in this study by additive W70 was due to reduced DMI rather than increased nutrient utilization.

The 17% CP diet improved DMI, milk yield, MUN, milk protein content, FCM yield, ECM yield, and feed efficiency (milk yield/DMI, FCM/DMI, and ECM/DMI) in this study. This aligns with findings that higher dietary protein levels enhance milk production and efficiency when adequately balanced with energy intake [30, 31]. Excessive protein intake without sufficient energy supply, however, may reduce these benefits, leading to increased MUN concentrations and lower nitrogen efficiency [32]. Milk yield and milk protein synthesis were enhanced by the 17% CP diet, likely due to improved AA availability and better MP supply. This aligns with previous findings that balancing MP levels can increase milk protein output by meeting mammary gland AA requirements [33]. According to the NASEM (2021) model, the nitrogen intake in the 17% CP diet was 102.4% of the estimated requirement in this study, indicating a sufficient supply to support milk yield and milk protein synthesis. In contrast, the nitrogen intake in the 16% CP diet was only 97.8% of the requirement in this study, creating a state of marginal protein deficiency that likely limited milk production potential. Although the 17% CP diet increased milk protein yield and concentration, milk fat and lactose yields remained unchanged, consistent with the view that dietary protein primarily affects protein synthesis without an impact on milk fat or lactose [34, 35]. In this study, FCM, milk yield/DMI, FCM/DMI, and ECM/DMI were higher under the 17% CP diet due to increased milk yield, while elevated milk protein content further contributed to the rise in ECM. MUN is a key indicator of NUE [36] and increases with higher dietary protein levels. In this study, MUN was increased with the 17% CP diet, consistent with findings that dietary CP is the primary predictor of MUN [37, 38].

### The effect of the additive W70 and dietary protein on rumen fermentation

There was no dietary protein and additive interaction effects on rumen fermentation indexes of lactating dairy cows. Probiotics can influence rumen fermentation patterns, including VFA production and profiles [39, 40], but additive W70 did not affect total VFAs, individual VFA, or the acetate-to-propionate ratio. This result aligns with studies showing that probiotics do not alter total VFA concentrations, even when altering the dietary ingredients [41, 42]. While other strains, such as *Lactobacillus plantarum*, lower NH₃-N without affecting pH or methane production [43], additive W70’s effects appear to be specific to nitrogen metabolism rather than general fermentation patterns [12]. The addition of additive W70 reduced ruminal NH₃-N concentrations in this study, indicating improved nitrogen utilization in the rumen. This reduction aligns with findings that probiotics enhance ammonia utilization by favouring microbial communities that efficiently convert NH₃-N into MCP [44, 45]. Lower NH₃-N levels are beneficial in high-protein diets, where excess nitrogen often leads to inefficiencies and potential health issues [46]. Although MCP is a critical protein source for cows, additive W70 did not increase MCP synthesis in this study. This might be due to additive W70 maintaining a balance in microbial activity rather than stimulating MCP production directly. Previous studies have noted that MCP synthesis often depends on fermentation conditions and dietary composition [17].

The 17% CP diet tended to reduce ruminal isovalerate and valerate concentrations, increase the acetate-to-propionate ratio, and elevate NH₃-N, while leaving ruminal pH unchanged in this study. Dietary CP concentrations generally do not affect ruminal pH in previous studies [47]. Although total VFAs and most individual VFAs (acetate, propionate, iso-butyrate, and butyrate) were unaffected, the higher CP diet altered the VFA profile by increasing acetate relative to propionate, potentially reflecting enhanced fermentation of fibrous carbohydrates [39]. Ruminal isovalerate, a byproduct of branched-chain AA deamination, showed inconsistent trends compared to prior studies, suggesting that dietary CP effects on rumen microbiota require further investigation [48]. The increase in NH₃-N with the 17% CP diet is consistent with studies showing a linear rise in NH₃-N with higher CP levels due to increased nitrogen availability from dietary protein breakdown [47, 49]. MCP production was unaffected by the 17% CP diet in this study, likely due to a balance between its production in the rumen and utilization in the small intestine, which highlights the nuanced role of dietary protein in rumen nitrogen metabolism and fermentation pathways.

### The effect of additive W70 and dietary protein on blood indicators

No dietary protein and additive interaction effects were observed for blood chemistry indexes in lactating dairy cows. Additive W70 supplementation did not affect albumin (ALB), globulins (GLB), total protein (TP), glucose (GLU), total cholesterol (T-CHO), triglycerides (TG), alanine aminotransferase (ALT), or aspartate aminotransferase (AST) levels, aligning with studies showing no changes in these parameters with probiotic supplementation [50–54]. BUN, a marker of nitrogen utilization [55], was unaffected by additive W70 supplementation in this study, though probiotics have been reported to reduce BUN by enhancing ruminal nitrogen utilization and limiting ammonia conversion to urea [51].

The 17% CP diet increased BUN and GLU levels, while ALB, GLB, TP, T-CHO, TG, ALT, and AST remained unchanged in this study. The increase in BUN reflects higher nitrogen absorption from dietary protein digestion, with excess nitrogen excreted via the kidneys [56, 57], which aligns with the role of BUN as a marker of nitrogen excretion efficiency. Higher GLU levels with 17% CP in this study suggest elevated gluconeogenic activity in the liver, where AA from dietary protein are converted into glucose to meet metabolic demands [58].

### The effect of additive W70 and dietary protein on nitrogen metabolism

A dietary protein and additive interaction effect was observed for N intake, retained N and its proportion, productive N and its proportion, and NUE in lactating dairy cows. The additive reduced N retention and productive N at 16% CP but no difference at 17% CP, suggesting protein-dependent effects. Interactions for N intake and NUE further indicate a stronger additive response under 16% CP conditions.

Additive W70 supplementation reduced N intake and improved NUE, largely due to reduced DMI in this study. This aligns with previous studies showing that lower N intake can enhance energy retention in ruminants by improving nutrient efficiency [59]. Despite the reduction in N intake, additive W70 supplementation had no effect on milk N, urinary N, fecal N, losses N, retained N, or productive N in this study. Probiotic effects on NUE typically involve either reducing nitrogen excretion or increasing nitrogen retention. For example, Lactobacillus-fermented products lower urinary N excretion, mitigating environmental impacts during early lactation [60]. Similarly, bacterial DFMs like *Bacillus amyloliquefaciens* H57 have improved N retention and reduced ruminal ammonia levels by altering rumen microbiota and feeding behaviour in ewes [29, 61]. In contrast, fungal DFMs such as *Saccharomyces cerevisiae* have demonstrated their ability to enhance feed efficiency and milk production by improving rumen fermentation and VFA production, although they often show minimal direct effects on N metabolism [62, 63]. These effects of Fungal DFMs are mediated by microbial shifts that optimize nutrient use and reduce losses.

The 17% CP diet increased milk N, N intake, fecal N, N loss, productive N, fecal N proportion, urine N proportion, N loss proportion, productive N proportion, and decreased NUE, while urinary N, retained N, and retained N proportion remained unchanged in this study. Higher dietary protein intake naturally led to increased N intake, milk N secretion and elevated fecal N content due to greater excretion of undigested protein, contributing to increased N losses. These findings align with previous research showing linear increases in MUN, BUN, milk N, and N excretion (fecal and urinary) as dietary CP levels rise from 11.9% to 16.2% [47]. Similarly, MP-sufficient diets result in higher BUN and urinary N excretion compared to MP-deficient diets, which is consistent with the results of this study [56]. However, low-protein diets, such as 16.1% CP, can maintain milk and milk protein yields while reducing N losses in urine and milk urea, highlighting their potential to mitigate nitrogen losses without performance trade-offs [34]. Adjustments in diet composition, such as reducing neutral detergent fiber (NDF) and increasing starch, can improve nitrogen utilization by promoting microbial protein synthesis in the rumen, even in lower-protein diets [31]. However, such strategies may not fully compensate for reduced protein levels, as shown by studies where reductions in CP content led to lower N losses and improved NUE but only marginal effects from starch or rumen-protected AA supplementation [37].

### The effect of dietary protein and additive W70 on rumen microbiota

At the genera level, the relative abundance of the genus *Sphingomonas*,* Solibacillus*,* Desemzia*,* Peribacillus*,* Succinivibrionaceae UGG-001*,* Prevotella*, and *Succiniclasticum* were higher in A than C, and the relative abundance of the Family p-251-o5 was lower in A than C. Similarly, the relative abundance of *Succinivibrionaceae*_UCG_001 was higher in high NUE cows than in low NUE cows [64]. The additive may have selectively promoted the enrichment of *Solibacillus*, which is potentially beneficial for host health [65, 66]. Similarly to this study, the genus *Prevotella* was higher in the rumen of Nellore steers with high feed efficiency (FE) phenotypes [67] and the ruminal copies of *Prevotella ruminicola* in high nitrogen utilization (HNU) phenotype goats were higher than those in the low nitrogen utilization (LNU) phenotype goats assessed using real-time PCR [68]. The addition of Bovine *Pichia kudriavzevii* T7, *Candida glabrata* B14, and *Lactobacillus plantarum* Y9 improve performance and regulate the dominant bacteria *Prevotella* in rumen and feces of dairy cows [69]. Previous studies also reported that rumen microbes (genera level) of *Prevotella* was associated with differentially expressed genes regulating RFI [70]. Additive W70 might have provided metabolites (e.g., lactate) that served as substrates for Prevotella, which can further metabolize them into propionate. *Peribacillus* [71, 72] and *Desemzia*, are belonging to the family of *Bacillaceae*. *Bacillaceae*, as spore-forming bacteria, which contribute to rumen function by producing enzymes like proteases, amylases, and cellulases, aiding in the breakdown of complex feed components and enhancing microbial activity [73]. *Bacillaceae* also enhanced rumen fermentation by stimulating volatile fatty acid production to improve energy availability for the host [74]. Based on additive W70 increasing the abundance of this taxa suggests an improvement in the nutrient utilising capacity of the rumen. At the genera level, the relative abundance of the genus *Kandleria*,* Ligilactobacillus*, and *Escherichia-Shigella* were lower in 17% than 16%, maybe inhibited by the higher CP diet in this study.

At the ASV level, ASV *Ruminococcus*, ASV *Xylanibacter*, ASV *Lachnospiraceae*, and ASVs NK4A214 increased under the addition of additive W70, indicating the fiber or hemicellulose hydrolysis [75, 76]. ASV *Prevotellaceae* and ASV Family F082 were increased, indicating that the succinate or acetate pathway becomes active, providing substrates for subsequent propionate production [67]. ASV *Sphingomonas*, ASV *Sphaerochaeta*, ASV *Psychococcus*, ASV *Solibacillus*, ASV *Jeotgalibaca* increased, indicating that diversified substrate hydrolysis and byproduct clearance, suggesting that additives may improve the micro-environment or inhibit competitors [77]. And also ASV *Solibacillus* is potentially beneficial for host health [65, 66]. At the ASV level, ASV *Butyrivibrio*, ASV *Alistipes*, ASV *Bacteroides*, and ASV *Sharpea* increased in the absence of additives, indicating that substrate utilization relied more on traditional fiber-butyrate fermentation and on protein- and lipid-oriented pathways [78–80], with ASV *Sharpea* acting as a strong lactic acid producer [81].

At the ASV level, the ASV *Lachnospiraceae* and ASVs associated with the genus *Xylanibacter* were increased, indicating an enhanced potential for the utilization of fiber or hemicellulose in roughage and the production of butyrate in 17% CP diet [76, 82, 83]. The enhancement of ASV *Muribaculaceae* and ASV [Eubacterium] ruminantium in 17% CP diet is also consistent with polysaccharide fermentation and butyrate or acetate supply [84, 85]. This may be due to that 17% dietary protein having increased nitrogen availability and promoting the growth requirements of fiber degrading bacteria. At the ASV level, the lactic acid bacteria and facultative bacteria (ASV *Kandleria*, ASV *Ligulactobacillus*, ASV *Escherichia-Shigella*) were increased in 16% CP diet, indicating that 16% dietary protein conditions increase soluble sugar dependence [86]. The increase in ASVs *Treponema* and ASV Family *Bacteroidales* in 16% CP diet may be due to the occupation of the ecological niche [87, 88].

Additive W70 may have engaged in cross-feeding or synergistic interactions with key rumen bacteria. F082 was positively correlated with iso-butyrate and total VFA in the additive supplementation group, and with acetate and total VFA in the 16% CP group. This indicated that F082 participated in fiber decomposition and the potential to generate precursors for branched-chain fatty acids. *Acinetobacter* was negatively correlated with NH_3_-N and MCP in the additive supplementation group, and with NH_3_-N in the 17% CP group. The pattern suggests that *Acinetobacter* may compete for nitrogen sources or be involved in metabolic pathways that reduce ammonia accumulation, thereby influencing the efficiency of nitrogen utilization. But this genus remains under-studied in the rumen context. NK4A214 was negatively correlated with the acetate-to-propionate ratio, and *Burkholderia Caballeronis Paraburkholderia* showed a negative correlation with iso-butyrate in the 17% CP group. The concurrent changes in these two taxa alongside key fermentation metrics suggest their coordinated role in modulating the ruminal fermentation pattern.

## Conclusion

Supplementing lactating cows with additive W70 improved feed conversion (milk yield/DMI) and nitrogen utilization efficiency, with the strongest response for the 16% CP diet. 17% CP diet increased milk and milk protein yields and improved FCM/DMI and ECM/DMI, but raised ruminal NH_3_-N, BUN, and total N excretion (fecal and urinary), indicating greater N losses. Overall, formulating 16% CP diet in combination with additive W70 emerges as a practical strategy to maintain or enhance efficiency while mitigating N losses.

## Methods

### Animal ethics

The study procedure and ethics were approved by the Animal Care Guidelines of the Chinese Academy of Agricultural Sciences Animal Care and Use Committee (No. IAS2022-91).

### Animals, diets, and experimental design

A statistical power analysis was carried out with an α = 0.05 and power = 0.80, and the sample size required with 0.4 effect size was a minimum of 13 cows, as estimated using G power 3.1 software. Therefore, sixteen early-lactation multiparous Holstein cows that were on average (mean ± SD) 137 ± 12.2 DIM, producing 29 ± 1.7 kg milk/d, and with 769 ± 15.2 kg body weight (BW) were used in the study. Cows were evaluated in a repeated 4 × 4 Latin with two levels of CP (16% vs. 17%) and two levels of additive W70 (0 g vs. 20 g/head per day). The cows were randomly assigned to a treatment sequence using the random function in Excel (Microsoft Corp., Redmond, WA, USA). Cows were assigned 4 treatment groups: 16% CP no additive (16%C), 16% CP with additive W70 (16%A), 17% CP no additive (17%C), 17% CP with additive W70 (17%A). Cows received 20 g/d of either whey powder used as control (16%C and 17%C) or freeze-dried additive *L. agilis* W70 (CGMCC 25654) (5 × 10^10^ cfu/g) (16%A and 17%A) during the treatment period. DFM preparations were administered top-dressed on the diets during the morning feeding. DFM viability was checked prior to each treatment period of the study by the viable plate count method.

The study began with a 14-day adaptation period for the dairy cows to acclimate to individual stalls, followed by a 7-day covariate period to collect baseline data on pre-experimental milk yield and total mixed ration (TMR) intake. Following this, the experimental phase of 28 days, consisting of a 7-day washout period without the DFM additive and a 21-day treatment period with the DFM additive was conducted. During the 21 d treatment periods, individual feed intake and milk yield of the cows were recorded daily. During the final three days of each experimental period, samples of milk, rumen fluid, fecal, and urine were collected.

Cows were housed together in a freestall barn with sand-bedded individual stalls. Cows were assigned to individual feeding positions according to the order of enrolment during the training period, when each cow was trained to eat from the assigned bunk. The experimental pens were equipped with fans. Low-pressure nozzles were mounted over the feeding bunk, facing away from the feed and onto the cows. Fans and nozzles were activated whenever ambient temperature reached 21 °C. Water was available ad libitum. Beddings were cleaned once a day and manure was flushed from the pen once daily via an automated flushing system.

The 2 experimental basal diets were formulated to satisfy nutrient requirements (NASEM, 2021; Table 6) using NASEM (2021) model and software (Eighth revised editionV8 R2022.01.18; National Academies of Sciences, Engineering and Medicine, NASEM Dairy 8). Cows were fed twice daily at 0600 and 1200 h, with 50/50% of the total daily allotment, respectively. Feed offered was adjusted daily based on the intake of the preceding 1 d and fed ad libitum with a minimum daily refusal ranging from 5 to 10%.

**Table 6:** Ingredient and chemical composition of the experimental diets fed to dairy cows (*n* = 16)

Item	16%^1^	17%^1^
Ingredient, % of DM		
Corn silage	35.50	35.50
Concentrate supplementation	18.41	18.41
Steam-flaked corn	18.26	15.94
Soybean meal	6.95	9.26
Wrapped silage	5.40	5.40
Imported alfalfa	4.63	4.63
Molasses	2.89	2.89
Cottonseed meal	1.78	1.78
Cottonseed	1.73	1.73
5% premix^2^	1.23	1.23
Fat meal	1.15	1.15
Beet grain	1.06	1.05
Sodium bicarbonate	0.58	0.58
Urea	0.38	0.38
Mold adsorbents	0.04	0.04
Nutrient composition, % of DM		
DM, % as fed	53.10	53.20
CP	16.10	17.00
RDP^3^, % CP	67.70	68.24
RUP^4^, % CP	32.30	31.76
RDP^5^	10.9	11.6
RUP^6^	5.2	5.4
MP^7^	9.23	9.66
NDF	29.30	29.40
ADF	18.80	18.90
Starch	24.70	23.00
NFC^8^	45.84	44.72
EE	0.96	0.98
Ash	7.8	7.9
Ca	0.47	0.48
P	0.47	0.48
NE_L_, Mcal/kg^9^	1.75	1.74

^1^16% = 16% CP diet, 17% = 17% CP diet

^2^Contained 15% Ca, 5% P, 4% Mg, 10% Na, 15% Cl, 0.2% K, 0.2% S, 2,900 mg/kg Fe, 1,860 mg/kg Mn, 510 mg/kg Cu, 260 mg/kg Zn, 45 mg/kg I, 25 mg/kg Se, 20 mg/kg Co, 332,000 IU/kg vitamin A, 88,000 IU/kg vitamin D, and 1,700 IU/kg vitamin E

^3–7^Estimated based on NASEM (2021)

^8^NFC = 100 − (FA + 1 + NDF + CP + ash)

^9^Calculated based on NASEM (2021) model and software (Eighth revised editionV8 R2022.01.18; National Academies of Sciences, Engineering and Medicine, NASEM Dairy 8)

### Feed intake and chemical analysis

The amount of feed offered and refused was measured and recorded for individual cows daily to estimate voluntary feed intake. Approximately 200 g of the feed from each cow was taken 3 times weekly, dried in a forced-air oven at 65 °C to constant weight. Dried TMR samples were composited on a weekly basis and stored for subsequent analysis. Composited TMR ingredients were ground in Wiley mills (A. H. Thomas Scientific, Philadelphia, PA, USA) to pass through a 1.0 mm sieve and stored for later analysis. Samples of TMR were analyzed for contents of dry matter (DM) (loss of weight after drying at 105℃, AOAC, 2000; method 930.15), CP (AOAC, 2000; method 984.13), NDF [89], acid detergent fiber (ADF) (AOAC, 2000; method 973.18), ether extract (EE) (AOAC, 2000; method 920.39), Ash (AOAC, 2000; method 942.05), Ca (AOAC, 2000; method 935.13), P (AOAC, 2000; method 965.17), estimated non-fiber carbohydrates (NFC) (NASEM 2021), estimated rumen degradable protein (RDP) (NASEM 2021), estimated rumen undegradable protein (RUP) (NASEM 2021), and calculated NE_L_ (NASEM 2021). Starch content was determined using a colorimetric method [90].

### Milk yield and milk components

#### Milk yield

Milk yield was recorded electronically in a voluntary milking system (DeLaval International AB, Tumba, Sweden) at each milking. Cows were milked three times at 700, 1300, and 2100 h, and a sample was collected and mixed in a 4:3:3 ratio (40 mL, 30 mL, and 30 mL) [91] in the last three days of the treatment period for composition analysis. Milk samples were preserved with 2‑bromo-2-nitropropane-1,3-diol (D&F Control Systems Inc., MA, USA), and stored at 4 °C.

#### Milk components

Milk samples were analyzed for milk fat, true protein, lactose, total solids, and MUN using a near-infrared reflectance spectroscopy analyzer (Foss Electric, Hillerød, Denmark). Somatic cell count was analyzed using a Fossomatic 5000 apparatus (Foss Electric, Hillerød, Denmark). Milk components were adjusted for the milk yield and used to calculate the daily component yield. The ECM yield was calculated as [(0.327 × milk yield) + (12.95 × milk fat yield) + (7.65 × milk protein yield)] (NRC, 2001). The 3.5% FCM yield was calculated as [(0.4324 × milk yield) + (16.216 × milk fat yield)] (NRC, 2001). Weekly mean values were calculated from the last three days values and used for statistical analysis.

### Ruminal fluid

#### Ruminal fluid collection

On the last day of the feeding trial of each period, the cows were transferred back to their pens, and ruminal fluid samples were collected 2 h before offering their diet in the morning, as described by Mesgaran et al. [92]. The rumen fluid were subjected to visual examination to ensure no saliva contamination was present. The rumen fluid samples suspected to be contaminated were removed, and fresh samples were taken.

#### pH, VFA, NH_3_-N, MCP, and methane

100 mL of rumen fluid was taken from each cow using a esophageal tube connected to a vacuum pump, and the rumen pH was immediately measured and read. 45mL of rumen fluid was placed in a plastic bottle containing 9 mL of 10% H_2_SO_4_ to prevent nitrogen volatilization and stored at -20 °C until analysis. The VFA contents of fermentation were determined by gas chromatography as described previously [93]. NH_3_-N was measured using an adaptation of the phenol/hypochlorite method [94]. MCP was determined by the Folin phenol method [95]. Estimated from stoichiometric calculation using the following equation: Methane = 0.45 × acetate (mol/100 mol) − 0.275 × propionate (mol/100 mol) + 0.40 × butyrate (mol/100 mol) [96].

### Blood

On the last day of the feeding trial of each period, the cows were transferred back to their pens, and blood samples were collected 2 h before feed was offered. 50 mL of blood was taken from the jugular vein of each cow and kept in a test tube containing EDTA, then centrifuged at 500 × g for 10 min to obtain plasma and kept at -20 °C for further analysis [97]. TP, ALB, GLB, BUN, GLU, T-CHO, and TG were assessed colourimetrically using the assay kit (Nanjing Jiancheng Bioengineering Institute Co., Ltd., Nanjing, China) according to the manufacturer’s protocol. ALT and AST were assessed colourimetrically using the assay kit (Beijing Boxbio Science & Technology Co., Ltd., Beijing, China) according to the manufacturer’s protocol.

### Nitrogen (N) metabolism

Apparent digestibility of DM and nutrients was determined using acid insoluble ash as an internal digestibility marker [98]. Fecal samples were collected during the 4 sampling periods. Fecal samples (approximately 400 g per sample) were collected from rectum of each cow 8 times in the last 3 d during each experimental period at the following times: 1000, 1600, and 2200 h (d 1), 0400, 1300, and 1900 h (d 2), and 0100 and 0700 h (d 3) as described in Oh et al. [62] and Lee et al. [56].

Fecal samples were pooled across 8 sampling times and divided into two portions. One portion was dried in a forced-air oven at 65℃ to a constant weight for the analysis of DM, OM, CP, NDF, and ADF, following the procedure described in method 2.3. The other portion was treated with 10 mL of 10% sulfuric acid per 100 g of fecal sample for nitrogen fixation, to determine fecal N. Apparent total-tract digestibility of DM, OM, CP, NDF, and ADF was estimated using acid insoluble ash (AIA) as an intrinsic marker [98].

200 mL urine samples were collected by palpation at the same time points as for fecal samples and processed as described in Oh et al. [62]. Samples were filtered through 2 layers of cheesecloth, and 20 mL of samples of urine were mixed with 80 mL of 10% H_2_SO_4_ and stored at -20 °C for urine N and creatinine analysis. Daily urine volume was estimated as BW × 29/urinary creatinine concentration (mg/L) [99].

Nitrogen retention was calculated as daily N in faeces, urine, and milk subtracted from daily N intake, as described in Gehman et al. [100]. Productive N was calculated as the sum of N retained in the body and N secreted in milk. The estimated apparent NUE percentage was calculated as NUE % = (kg milk N/kg dietary N intake) × 100.

### 16S rRNA gene amplicon sequencing of rumen microbiota

High-throughput sequencing of the V3-V4 hypervariable region of the bacterial 16S rRNA gene was performed on an Illumina MiSeq platform according to their standard protocols (Beijing Genomics, Beijing, China). Briefly, the V3-V4 region was PCR-amplified using universal primers containing adapter overhang nucleotide sequences for forward and reverse index primers. Amplicons were purified using AMPure XP beads (Beckman Coulter, Indianapolis, IN, USA) and set up for the index PCR with Nextera XT index primers (Illumina, San Diego, CA, United States). The indexed samples were purified using AMPure XP beads, quantified using a fragment analyzer (Agilent, Santa Clara, CA, USA), and equal quantities from each sample were pooled. Single-stranded PCR products were produced via denaturation. The reaction system and program for circularization are subsequently configured and set up. Single-stranded cyclized products are produced, while uncyclized linear DNA molecules were digested. Single-stranded circle DNA molecules are replicated via rolling cycle amplification, and a DNA nanoball (DNB) which contains multiple copies of DNA is generated. Sufficient quality DNBs are then loaded into patterned nanoarrays using high-intensity DNA nanochip technique and sequenced through combinatorial Probe-Anchor Synthesis (cPAS). The resulting pooled library was quantified using the Bioanalyzer 7500 DNA kit (Agilent) and sequenced using the V3-V4 chemistry (2 × 300 bp paired-end reads). The library was prepared using a 2 × Phanta polymerase Max Master Mix (VAZYME, Nanjing, China), and the V3-V4 variable region of 16S rRNA gene of bacteria was amplified by forward and reverse PCR degenerate primers F and R (338F: 5’-ACTCCTACGGGAGGCAGCAG-3’, 806R: 5’-GGACTACHVGGGTWTCTAAT-3’). PCR enrichment was performed in a 50 µL reaction containing 30ng template and fusion PCR primers. PCR cycling conditions were as follows: 95 °C for 3 min; 30 cycles of 95 °C for 15 s, 56 °C for 15 s, 72 °C for 45 s, and final extension at 72 °C for 5 min. PCR products were purified by DNA magnetic beads (BGI, LB00V60). The validated libraries were used for sequencing on Illumina MiSeq platform (BGI, Shenzhen, China) following the standard pipelines of Illumina, generating 2 × 300 bp paired-end reads.

Raw data was filtered to generate high quality clean reads according to the method of He et al. [101]. Truncated reads whose average phred quality values are lower than 20 over a 30 bp sliding window were truncated using iTools Fqtools fqcheck (v.0.25). Remove were filtered to discard those with trimmed lengths below 75% of the original, adapter-contaminated reads (Cutadapt v.2.6), reads with ambiguous base (N), and low-complexity reads (readfq v1.0).

Amplicon Sequence Variants (ASVs) were inferred using the DADA2 (Divisive Amplicon Denoising Algorithm) pipeline implemented in R software (v4.4.3). ASVs represent exact biological sequences with single-nucleotide resolution, offering greater taxonomic accuracy compared to traditional OTUs clustered at a fixed similarity threshold (e.g., 97%). In this study, the quality-filtered paired-end reads were first imported into QIIME2. The QIIME DADA2 denoise-paired function was then used to perform denoising, remove chimeric sequences, and construct a feature table based on the inferred ASVs. Finally, the feature table was exported in a standard format for downstream analysis and visualization.

ASV representative sequences are aligned against the Silva 16 S database (v138.2) for taxonomic annotation using the RDP classifier (v2.14) software (sequence identity was set at 0.6). The α-diversity of the microbial communities was measured using Hill numbers with q values of q0 (equivalent to species richness), q1 (equivalent to Shannon’s index) and q2 (equivalent to inverse Simpson’s index), which differ in how they weight rare ASVs (Amplicon sequence variants). Hill numbers were calculated from raw read counts using rarefaction/ extrapolation curves to account for the unequal sequencing depth [102]. The differences in α-diversity between the treatment groups was determined using linear mixed-effects models, with the main effect of Protein (16%-17%), main effect of Additive (A-C), and Period as fixed effects, and Cow identity as a random effect. β-diversity was calculated using the Bray-Curtis distance metric on relative abundance data. A PERMANOVA was then used to determine whether the microbial composition of the samples differed between the treatment groups. Permutations were restricted to only occur within individual cows, to account for the fact that there are multiple samples per individual, and in total 9999 permutations were performed. The differential abundance analysis method of DESeq2 [103] was used and analysed at both the ASV and genera level. Spearman rank correlation coefficients were used to calculate the correlation between the genera and the fermentation parameters, with the Benjamini-Hochberg method used to control for multiple tests.

### Statistical analysis

All data were analyzed using SAS version 9.3 (2003; SAS Institute Inc., Cary, NC, USA). Outliers were removed with the REG procedure based on an absolute studentized residual value > 3, and one dairy cow was removed from the last three periods of the trial due to an *Actinomyces* infection. Data were tested for normality using the UNIVARIATE procedure. All data were analyzed using the MIXED procedure.

Data for lactation performance, rumen fermentation, blood chemistry parameters, and N metabolism were analyzed by ANOVA Latin square. The model used was as follows:

$$\begin{aligned}{Y_{ijk12}} = & {\text{ }}\mu {\text{ }} + {\text{ }}{S_i} + {\text{ }}C{\left( S \right)_{ij}} + {\text{ }}{P_k} \\ &+ {\text{ }}{T_1} + {\text{ }}{T_2} + {\text{ }}{T_{12}} + {\text{ }}{E_{ijk12}}, \\ \end{aligned} $$

Yi_jk12_ is the dependent variable, µ is the overall mean, S_i_ is the random effect of square, C(S)_ij_ is the random effect of cow within square, P_k_ is the fixed effect of period, T_1_ is the fixed effect of DFM (control, additive W70), T_2_ is the fixed effect of dietary protein level (16% CP, 17% CP), T_12_ is the interaction between the dietary protein level and the dose of the additive, and E_ijk12_ is the residual error.

For the repeated measures analysis of dry matter intake, milk yield, milk composition, and feed efficiency, several covariance structures, including compound symmetry, first-order autoregressive [AR(1)], and unstructured were compared. The model with the AR(1) covariance structure was selected as it resulted in the lowest values for both Akaike’s information criterion (AIC) and Bayesian information criterion (BIC).

Where the main effect of treatment was significant, means were separated by pairwise t-test (pdiff option of PROC MIXED). Statistical differences were declared at *P* < 0.05. Differences between treatments at 0.05 ≤ *P* < 0.10 were considered as a trend toward significance. Data are presented as least squares means.

## Supplementary Information

Below is the link to the electronic supplementary material.

Supplementary Material 1

## Data Availability

The Illumina sequencing raw data for our samples are available in the NCBI Sequence Read Archive (SRA) under accession numbers: PRJNA1269080 in https://www.ncbi.nlm.nih.gov/search/all/?term=PRJNA1269080.

## Abbreviations

ADF: Acid detergent fiber
AIA: Acid insoluble ash
AIC: Akaike’s information criterion
ALB: Albumin
ALT: Alanine aminotransferase
AA: Amino acid
AST: Aspartate aminotransferase
BIC: Bayesian information criterion
BUN: Blood urea nitrogen
BW: Body weight
CP: Crude protein
DFMs: Direct fed microbials
DM: Dry matter
DMI: Dry matter intake
EAA: Essential amino acids
ECM: Energy corrected milk
EE: Ether extract
FCM: Fat corrected milk
FE: Feed efficiency
GLB: Globulins
GLU: Glucose
LAB: Lactic acid bacteria
MCP: Microbial protein
MP: Metabolizable protein
MUN: Milk urea nitrogen
NDF: Neutral detergent fiber
NFC: Non-fiber carbohydrates
NH_3_-N: Ammonia nitrogen
NUE: Nitrogen utilization efficiency
ASVs: Amplicon sequence variants
RDP: Rumen degradable protein
RUP: Rumen undegradable protein
T-CHO: Total cholesterol
TG: Triglycerides
TMR: Total mixed ration
TP: Total protein
VFAs: Volatile fatty acids
