# Supplementary material for: Effects of Ligilactobacillus agilis W70 and dietary protein levels on feed efficiency, nitrogen metabolism, and rumen microbiota of lactating dairy cows
Source: Anim Microbiome. 2026 Mar 24;8:58. doi: 10.1186/s42523-026-00548-7 (PMC13134307; doi:10.1186/s42523-026-00548-7)
Supplement: Supplementary file 1 — Supplementary Material 1 [file 42523_2026_548_MOESM1_ESM.pdf]

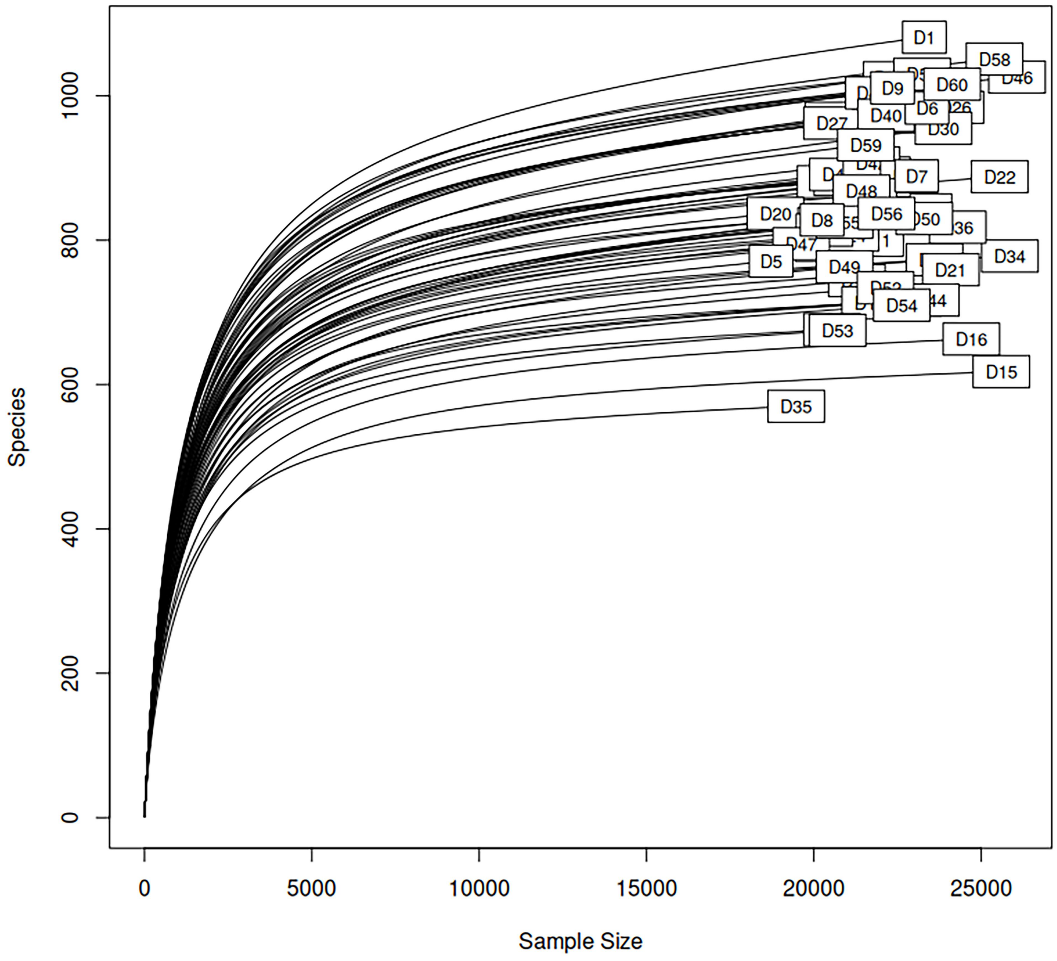

Fig. S1 Rarefaction curves of all 60 Samples.

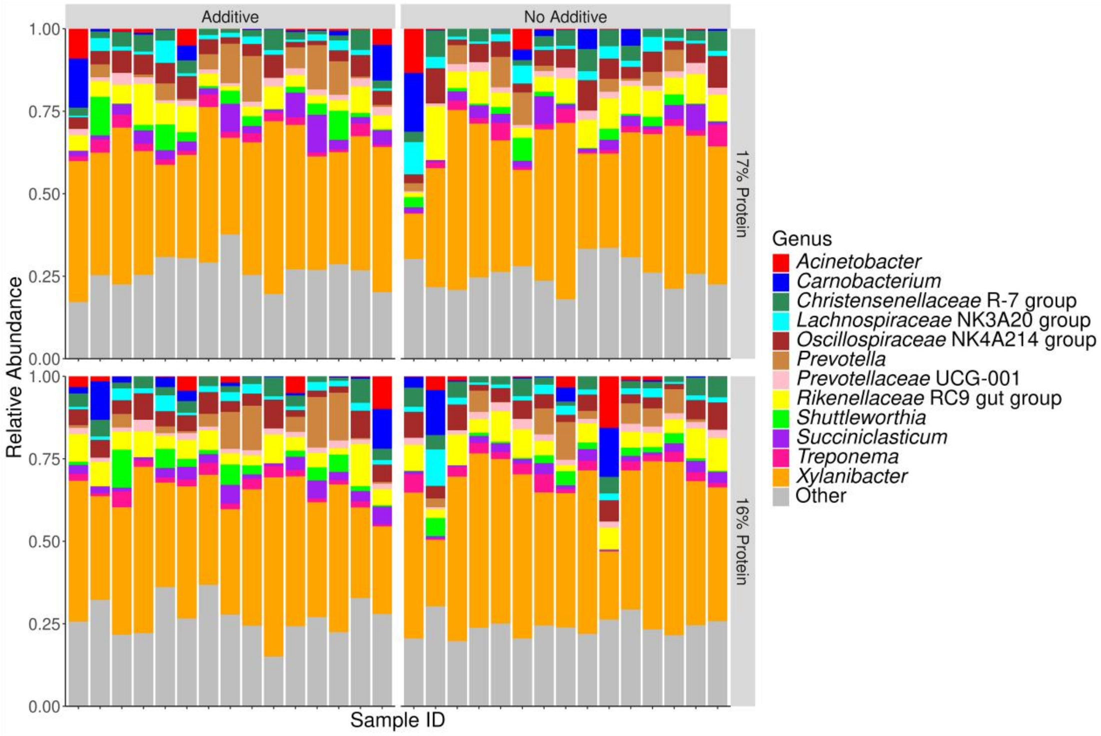

Fig. S2 Histogram of clustering of family abundance in main effects Additive (A-C) and Protein (16%-17%). (A = *L. agilis* W70 additive, C = no additive, 16% = 16% CP diet, 17% = 17% CP diet).

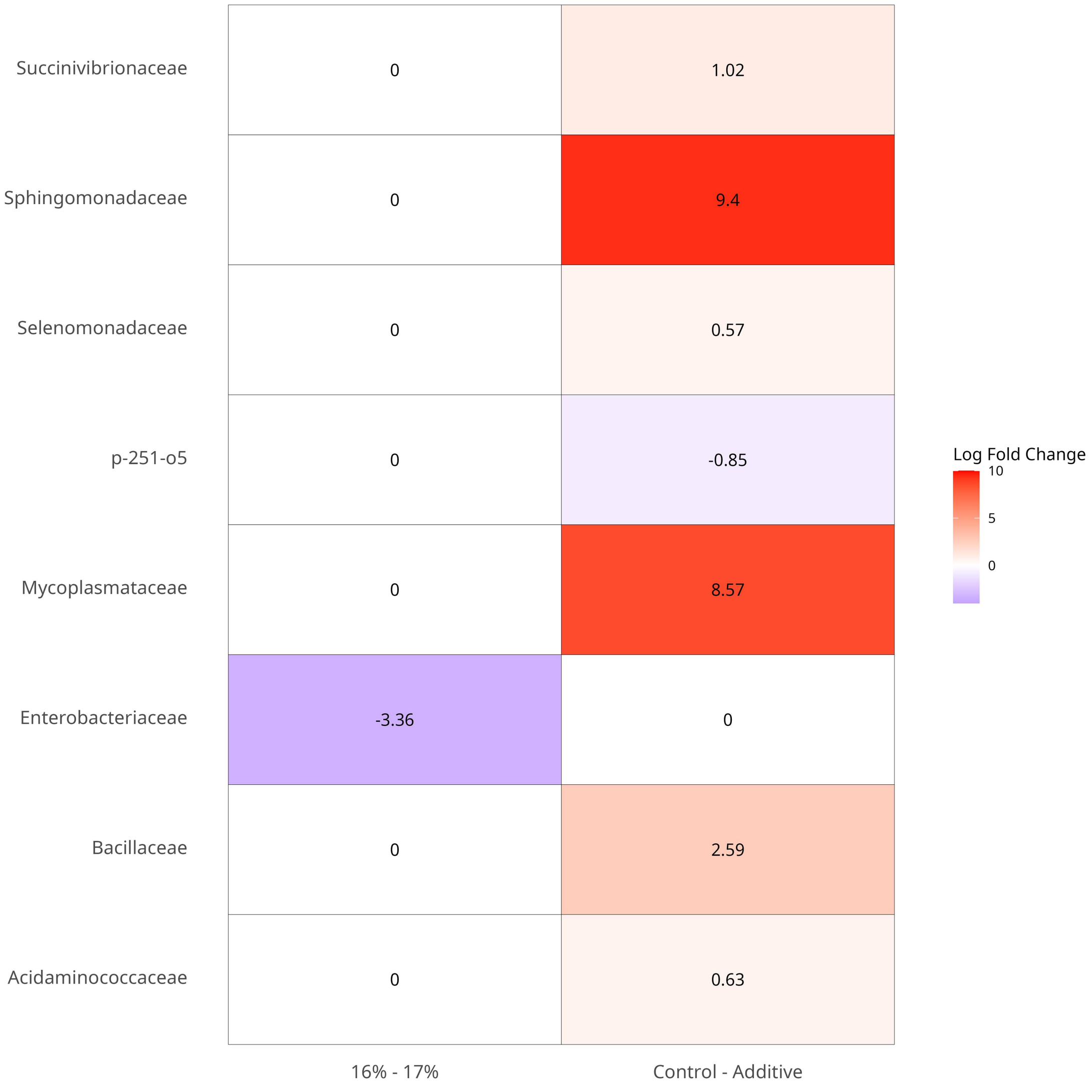

Fig. S3 DESeq2 analysis of relative abundance differences (adjusted *P*-value < 0.05) at the family level in main effects Additive (A-C) and Protein (16%-17%). (Negative log fold change estimates correspond to reduced abundance of each ASV in the 16% (left plots) and Control (right plots)) (A = *L. agilis* W70 additive, C = no additive, 16% = 16% CP diet, 17% = 17% CP diet).

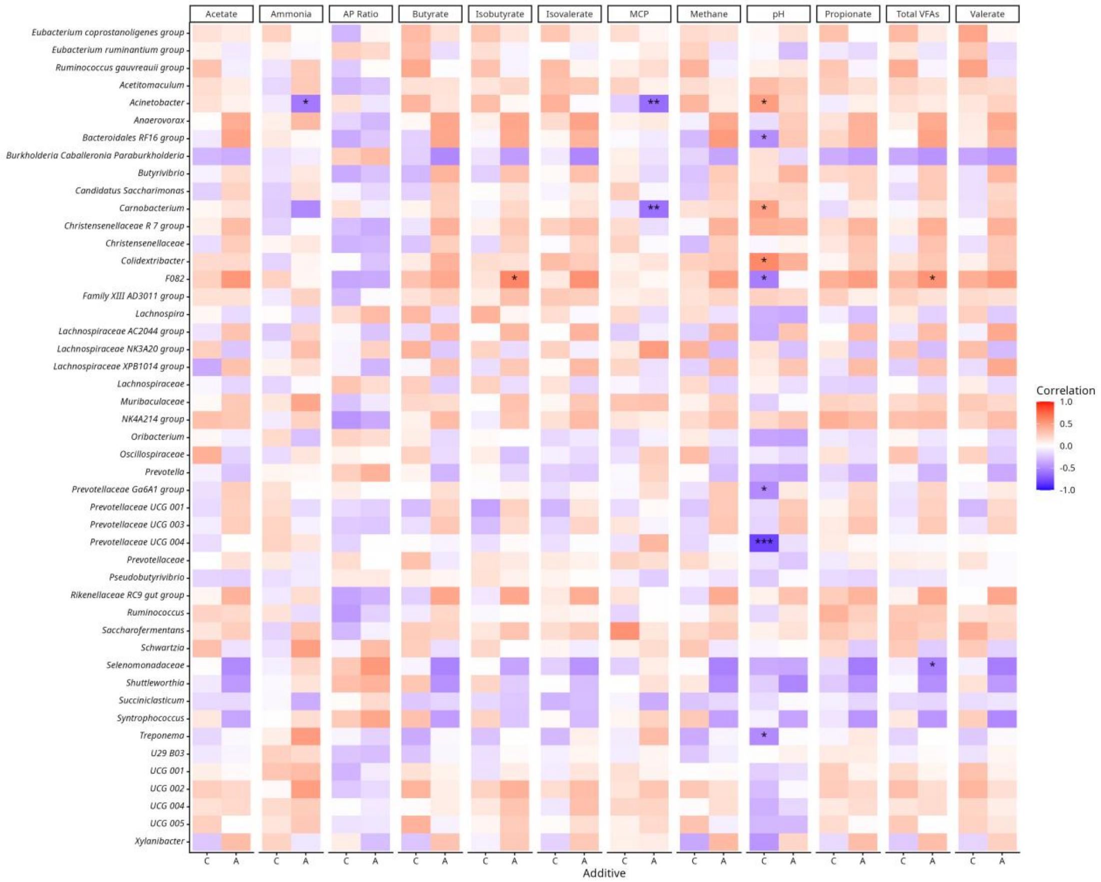

Fig. S4 Correlation analysis between rumen fluid microbiota and rumen fermentation parameters in main effects Additive (A-C). (Red indicates positive correlation, blue indicates negative correlation, white indicates no correlation, ***P < 0.001, **P < 0.01, *P < 0.05. A = *L. agilis* W70 additive, C = no additive).

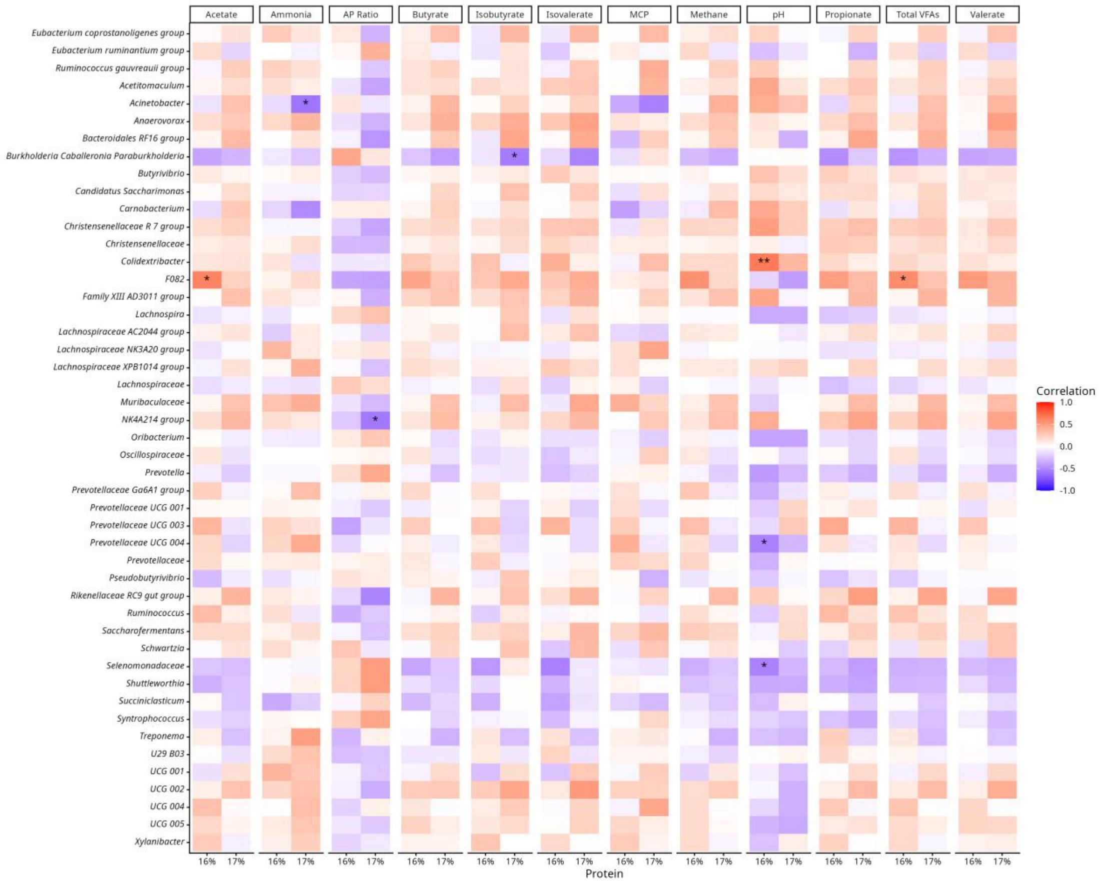

Fig. S5 Correlation analysis between rumen fluid microbiota and rumen fermentation parameters in main effects Protein (16%-17%). (Red indicates positive correlation, blue indicates negative correlation, white indicates no correlation, ***$P < 0.001$, **$P < 0.01$, *$P < 0.05$. 16% = 16% CP diet, 17% = 17% CP diet).

Table S1 The time effect and additive × time interaction effect *P*-values in parameters of lactation performance, rumen fermentation parameters, blood, and nitrogen metabolism

Item	*P* Value	
	Time	A×T[1]
DMI (kg/d)	<0.01	0.61
Production (kg/d)		
Milk yield	<0.01	0.27
Fat	<0.01	0.06
Protein	<0.01	0.63
Lactose	<0.01	0.14
3.5% FCM	<0.01	0.54
ECM	<0.01	0.60
Composition (%)		
Fat	<0.01	0.03
Protein	0.34	0.71
Lactose	<0.01	0.09
Total Solids	<0.01	0.26
Somatic cell count (10^4 cell/mL)	<0.01	0.60
MUN (mg/dL)	<0.01	0.61
Efficiency		
Milk yield/DMI	<0.01	0.63
FCM/DMI	<0.01	0.37
ECM/DMI	0.01	0.45
pH	<0.01	0.79
Total VFAs (mmol/L)	<0.01	<0.01
Acetate (mmol/L)	0.02	<0.01
Propionate (mmol/L)	0.03	<0.01
Iso-butyrate (mmol/L)	<0.01	0.48
Butyrate (mmol/L)	<0.01	0.01
Isovalerate (mmol/L)	<0.01	0.07
Valerate (mmol/L)	<0.01	0.01
A:P	0.12	0.02
Methane	<0.01	0.01
NH_3N (mg/dL)	<0.01	0.36
MCP (g/L)	<0.01	0.15
TP (g/L)	0.05	0.75
ALB (g/L)	0.15	0.44
GLB (g/L)	0.02	0.62

ALB/GLB	<0.01	0.37
BUN (mmol/L)	<0.01	0.71
GLU (mmol/L)	<0.01	0.62
T-CHO (mmol/L)	<0.01	0.67
TG (mmol/L)	0.41	0.17
ALT (U/mL)	<0.01	0.06
AST (U/mL)	<0.01	0.10
N Intake (g/d)	<0.01	0.01
Milk N (g/d)	<0.01	0.51
Fecal N (g/d)	<0.01	0.04
Urine N (g/d)	<0.01	0.78
Loss N (g/d)	0.03	0.90
Retained N (g/d)	0.02	0.04
Productive N (g/d)	0.03	0.01
Fecal N (%)	<0.01	0.03
Urine N (%)	<0.01	0.55
Loss N (%)	0.09	0.08
Retained N (%)	0.02	0.07
Productive N (%)	0.09	0.08
NUE	0.02	0.21

[1]A × T = interaction of *L. agilis* W70 addition and time.
